# Sinisan Alleviates Stress-Induced Intestinal Dysfunction and Depressive-like Behaviors in Mice with Irritable Bowel Syndrome by Enhancing the Intestinal Barrier and Modulating Central 5-Hydroxytryptamine

**DOI:** 10.3390/ijms251910262

**Published:** 2024-09-24

**Authors:** Haizhou Zeng, Yupeng Jiang, Qiuxiong Yin, Xinran Li, Yanli Xiong, Boyi Li, Xiaoying Xu, Huimei Hu, Guoqiang Qian

**Affiliations:** School of Chinese Medicine, Guangdong Pharmaceutical University, Guangzhou 510006, China; zenghaizhou1224@163.com (H.Z.); jiangyupeng404@163.com (Y.J.); yinqiuxiong163@163.com (Q.Y.); xinran202302@163.com (X.L.); xiongyanli106@163.com (Y.X.); liboyi201@163.com (B.L.); xxyeve@163.com (X.X.); h2huimei@163.com (H.H.)

**Keywords:** sinisan, diarrhea-predominant irritable bowel syndrome, inflammation, depression, TLR4/Myd88/NF-κB signaling pathway, brain–gut axis

## Abstract

Irritable bowel syndrome (IBS) is a common chronic functional bowel disorder and is strongly associated with an increased risk of depression and anxiety. The brain–gut axis plays an important role in the pathophysiologic changes in IBS, yet effective treatments for IBS are still lacking. Sinisan, originating from the *Treatise on Typhoid Fever* by the medical sage Zhang Zhongjing, is a classic formula in the Eight Methods of Traditional Chinese Medicine (TCM) that focuses on dispersing the liver and regulating the spleen, relieving depression and transmitting evils, and has been widely used in the treatment of liver-depression and spleen-deficiency, diarrhea, and related liver and stomach disorders. However, the therapeutic effect of sinisan in IBS has not been clarified. The aim of this study was to investigate the effects of sinisan on stress-induced intestinal dysfunction and depressive behavior in IBS mice. We established a diarrhea-predominant irritable bowel syndrome (IBS-D) mouse model using a 4% acetic acid enema combined with restraint stress, and analyzed the results using behavioral tests, relevant test kits, hematoxylin-eosin (HE) staining, immunofluorescence (IF), Western blot (WB), and quantitative fluorescence polymerase chain reaction (qRT-PCR). The results showed that sinisan administration significantly alleviated intestinal dysfunction and depressive-like behaviors in IBS-D mice, improved mild colonic inflammation and intestinal mucosal permeability, up-regulated the expression of tight junction proteins ZO-1 and occludin. Sinisan significantly alleviated intestinal dysfunction and depressive-like behaviors in IBS-D mice by decreasing the expression of TNF-α, promoting the expression of tight junction proteins (occludin, ZO-1) expression, and inhibiting the Tlr4/Myd88 signaling pathway, thereby attenuating the inflammatory response, protecting the intestinal barrier, and alleviating symptoms in the IBS-D mouse model. Taken together, sinisan may ameliorate intestinal inflammation and the intestinal barrier by regulating 5-HT expression and the Tlr4/Myd88 pathway, thereby alleviating stress-induced intestinal dysfunction and depressive behaviors in IBS-D mice.

## 1. Introduction

Irritable bowel syndrome (IBS) is a common chronic functional gastrointestinal disorder characterized by recurrent abdominal pain and changes in stool character and stool frequency. The pathogenesis of IBS is triggered by factors including intestinal motility disorders, visceral hypersensitivity, altered central nervous system mechanisms, and intestinal infections [1]. Recent studies have shown that the development of IBS is closely related to psychological factors, such as depression, anxiety-like mood and somatization disorders, which also contribute to the development of IBS patients, and that severe psychosocial problems are evident in about 60% of IBS patients [2]. In recent years, with the change in office patterns, global recession and accelerated social development, people’s social stress has been increasing day by day. The resulting incidence of IBS has increased significantly worldwide. The prevalence of the disease is about one fifth of the global population [3] and mainly affects adult females and adolescents with psychosocial co-morbidities [4]. It imposes a huge burden on people’s health and society’s medical resources, which seriously reduces work efficiency and affects patients’ living standards [5]. According to the Rome IV criteria, which were the most reported in the Rome Foundation’s global research findings, IBS can be divided into four types: diarrhea, constipation, mixed, and indeterminate, with diarrhea-predominant irritable bowel syndrome (IBS-D) being the most common, accounting for one-third of IBS cases [6].

The pathogenesis of IBS-D is unclear and may involve factors related to abnormal gut–brain interactions, stress, gut–brain axis dysfunction, disruption of gut barrier function, abnormal gastrointestinal motility, disruption of the gut microbiome, abnormal visceral hypersensitivity, intestinal inflammation and immune activation [7]. There is no specific treatment for IBS-D. Clinical treatment consists of serotonin receptor antagonists, dietary modifications, probiotics, and oral medications such as anticholinergics and 5-hydroxytryptamine (5-HT) agonists. Drugs approved by the U.S. Food and Drug Administration for the treatment of IBS-D include alosetron, eluxadoline, and rifaximin [8]. However, the symptoms often cannot be effectively controlled, and the long-term use of the above drugs may cause serious adverse reactions. Currently, the existing clinical treatments mainly treat IBS-D by treating the symptoms manifested by IBS-D, and there is a lack of more comprehensive, safe, effective treatments with fewer side effects [9]. Therefore, studying its pathogenesis and searching for effective clinical drugs are crucial for improving clinical outcomes and enhancing patients’ quality of life.

Chinese herbal medicine has a history of more than 3000 years in China, and Chinese herbal formulas have multi-component, multi-pathway, multi-target synergistic regulatory effects, which are unique and effective in the field of treatment of chronic disease prevention and control and epidemic prevention and control. Although they still belong to the category of complementary therapies, studies have found that Chinese herbal medicines have great potential, and the mechanism of their action and the enhancement of their therapeutic efficacy in IBS-D are of great significance [10,11]. Modern clinical studies have widely used Chinese herbal formulas such as Baizhu shaoyao Tang, Tong Xie yao Fang, and Shenlingbaizhu San in relieving the symptoms of IBS-D and have been widely used in China [12,13,14]. However, these remedies only treat IBS from the direction of gastrointestinal diseases, but lack the psychological factors to treat IBS at the same time. The theory of TCM on the pathogenicity of emotions is very similar to the modern theory of emotional stress, which suggests that the main pathogenesis of IBS is a series of disorders of the spleen and gastric functioning due to liver-qi stagnation and unfavorable detoxification, which is called “liver-stagnation and spleen-deficiency”. The main pathogenesis of IBS-D is liver depression and spleen deficiency [9], While sinisan is a representative formula for detoxifying the liver and harmonizing the liver and spleen, it is from the “*Treatise on Febrile Diseases*” by Zhang Zhongjing of the Eastern Han Dynasty, sinisan is composed of *Bupleurum chinense DC (chaihu), Paeonia lactiflora Pall (baishao), Citrus aurantium L. (zhishi)* and *Glycyrrhiza uralensis Fisch (gancao)* (all names have been checked at http://www.worldfloraonline.org). Modern studies have shown that sinisan has antidepressant, sedative–hypnotic, hepatoprotective, and anti-ulcer effects, and has been widely used clinically in the treatment of IBS [15,16,17,18]. However, the definitive mechanism by which sinisan affects IBS-D patients is currently unknown. Through our previous study, we found that sinisan significantly restored the impaired intestinal barrier function in a water-immersion restraint-stress mouse model by decreasing the serum levels of TNF-α, IL-1, IL-6 and other related inflammatory factors, and by modulating the disruption of the bacterial flora in the intestinal tract [19] (Xu et al., 2023), which further provides new insights into the potential mechanisms underlying sinisan treatment of diseases similar to those induced by restraint stress. In the present study, we explored the effects of sinisan on IBS-D induced by acetic acid enema combined with stress binding and elucidated the underlying mechanisms through in vivo studies.

## 2. Results

### 2.1. Identification of Compounds in the HPLC-MS/MS Analysis of Sinisan and Chromatograms

**Figure 1:**
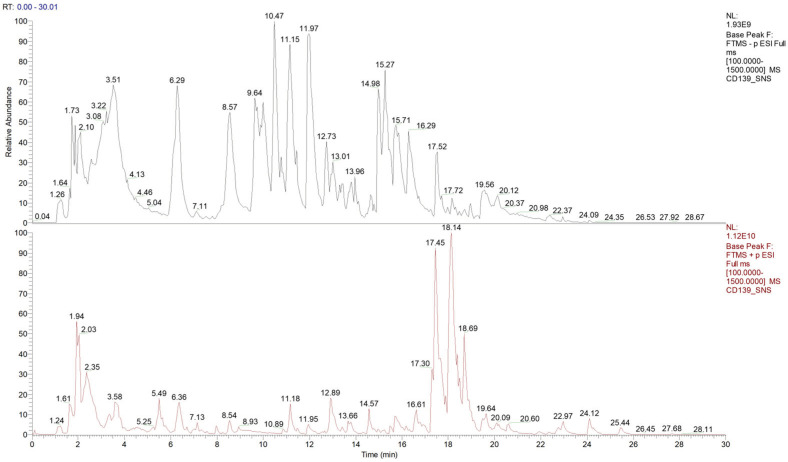
Total ion flow mapping for natural product identification in sinisan. Positive and negative mode analysis of sinisan in positive and negative ion mode using UHPLC/MS in the range of 100.0 to 1500.0 m/z. UHPLC-Q-TOF/MS information was obtained for all compounds and completed using CD 3.3 (Compound Discoverer 3.3) (Thermo Fisher, Waltham, MA, USA). The data were preliminarily organized and then searched in a database (mzCloud) for comparison. Note: Column 1 in black is the total ion flow map in negative ion mode. Column 2 in red is the positive ion mode total ion flow plot.

As shown in Figure 1, analyzing the precursor ions and fragmentation information of the peaks in the total ion chromatogram of the sinisan decoction, we matched a total of 955 compounds in mzCloud, and 505 compounds in the mzCloud best match had a composite score greater than 60. The compounds included chaihu saponin, paeoniflorin lactone glycoside, isoglycyrrhizin, 18β-glycyrrhetinic acid, tangerine, hesperidin, naringenin, naringenin chalcone, and other compounds. Related ingredients is shown in Supplementary S1.

### 2.2. Sinisan Ameliorates Intestinal Symptoms and Depression-like Behavior in IBS-D Mice

**Figure 2:**
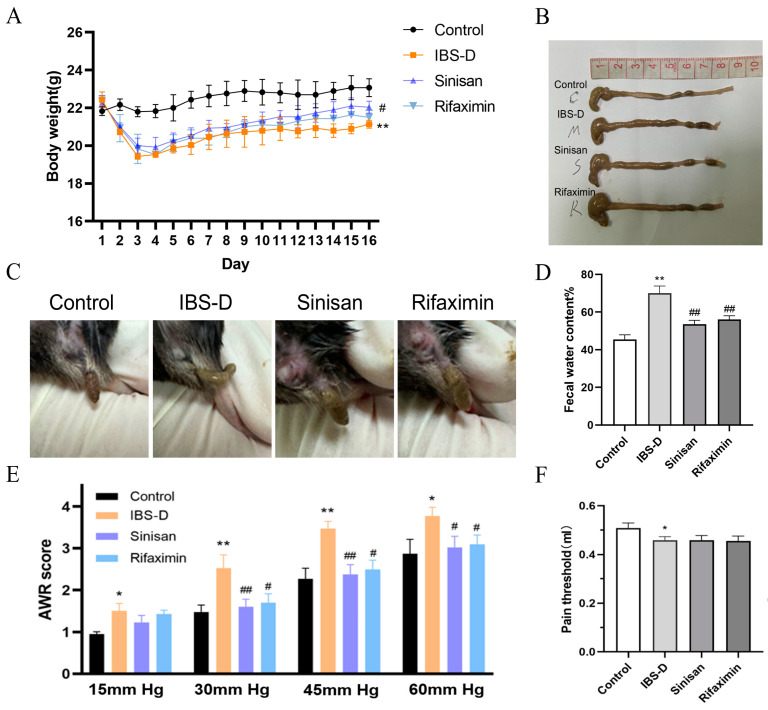
Sinisan relieved intestinal symptoms in IBS-D mice. (**A**) Body weight. (**B**) Representative gross anatomy of the mouse colon and colonic lengths. (**C)** Representative pictures of mouse feces. (**D**) Fecal water content. (**E**) AWR score. (**F**) Pain threshold. (*n* = 6), * *p* < 0.05, ** *p* < 0.01 compared with the control group. # *p* < 0.05, ## *p* < 0.01 compared with the IBS-D group.

In this experiment, we first established the IBS-D mouse model by acetic acid enema combined with stress restraint, and observed the changes in body weight, colonic lengths, fecal diarrhea, and visceral hypersensitivity reactions in different groups. The results showed in Figure 2 that the IBS-D group mice exhibited significantly lower body weight gain, lower colorectal pain threshold and persistent diarrhea after modeling, increased fecal moisture content and abdominal withdrawal reflex (AWR) score compared with the control group mice. No significant pathologic changes in colon were observed in all groups, confirming the successful construction of the IBS-D mouse model. After treatment, mice showed improvement in diarrhea, fecal water content, pain threshold and AWR score. There was no statistically significant difference between the rifaximin and sinisan groups. It indicated that sinisan improved the intestinal symptoms of IBS-D mice.

**Figure 3:**
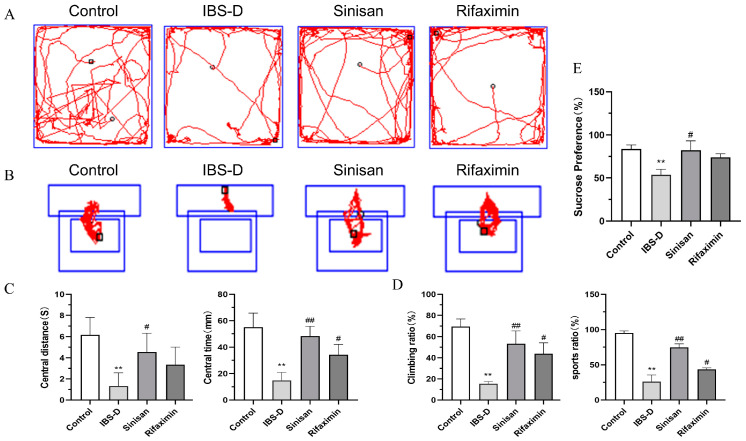
Sinisan alleviates depressive-like behavior in IBS-D mice. (**A**) Representative traces of locomotor activity in the OFT. Circles represent the activity of beginning and the squares represent the end. (**B**) Representative traces of locomotor activity in the TST. Squares represent the suspension positions during the experiment. (**C**) OFT center travel distance and center dwell time. (**D**) TST climb and sport rates. (**E**) Sucrose preference. (*n* = 6), ** *p* < 0.01 compared with the control group. # *p* < 0.05, ## *p* < 0.01 compared with the IBS-D group.

It is easy to overlook that stress induced by excessive tension and other psychological factors are important factors contributing to IBS-D. We explored the effects of sinisan and rifaximin on stress-induced IBS-D through behavioral experiments. As results in Figure 3 shown, compared with the control group, in the open field test (OFT), IBS-D mice passed less time and travelled shorter distances in the central region. In the tail suspension test (TST), IBS-D mice climbed and struggled for a shorter time. Meanwhile, IBS-D mice had less preference for 2% sucrose in the sucrose preference test (SPT). The sinisan and rifaximin treatments improved the depressive-like behavior, increased motion range, exercise-duration and sucrose preference in IBS-D mice, suggesting that sinisan can improve the depressive-like behavior of IBS-D mice more effectively than rifaximin.

### 2.3. Sinisan Ameliorated the Pathological Changes in IBS-D Mice

**Figure 4:**
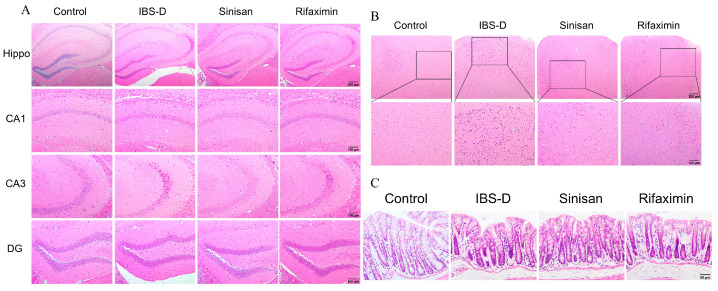
Sinisan ameliorated the pathological changes in IBS-D mice. (**A**) Hippocampal sections of the CA1, CA3, and DG regions were obtained and stained with HE (magnification 100× or 200×, scale bar = 200 μm or 100 μm). Hippo, hippocampus. (**B**) Prefrontal cortex sections (magnification 100× or 200×, scale bar = 200 μm or 100 μm). (**C**) Colon sections (magnification 40×, scale bar = 50 µm). (*n* = 6).

Depression-like behavior may lead to neuronal damage in the brain, which was observed in the hippocampal area and prefrontal cortical area by HE staining. Through HE staining to observe in the hippocampal area and prefrontal cortical area, we suppose that depression-like behavior may lead to neuronal damage in the brain. As shown in Figure 4, the HE staining results showed that hippocampal neurons in the control group had a normal morphology with clear boundaries, while the IBS-D group showed an abnormal morphology of neuronal atrophy, deepened nuclear staining, and even neuronal deletion. Sinisan treatment can alleviate this neuronal damage to some extent. In addition, sinisan treatment effectively suppressed the IBS-D-induced neuron reduction in the CA1, CA3 and dentate gyrus (DG) regions of the hippocampus. These results suggest that sinisan was able to attenuate IBS-D-induced neuronal death in the CA1, CA3 and DG regions of the hippocampus.

Regarding the histological structure of mouse colonic tissues, the control group had smooth and intact colonic mucosal structure, with sufficient epithelial cells, cup cells and glands, and normal morphology of the muscularis propria, with no inflammatory cell infiltration. In contrast, IBS-D mice had enlarged colonic intestinal glandular spaces accompanied by inflammatory cell infiltration and mild mucosal edema. Sinisan and rifaximin treatment alleviated the situation, with regular arrangement of mucosal glands, reduced edema and inflammatory cells. Sinisan effectively alleviated IBS-D related symptoms and promoted histologic recovery.

### 2.4. Effect of Sinisan on Serum Levels of Inflammatory Factors and Oxidative Stress in Colonic Tissues of IBS-D Mice

**Figure 5:**
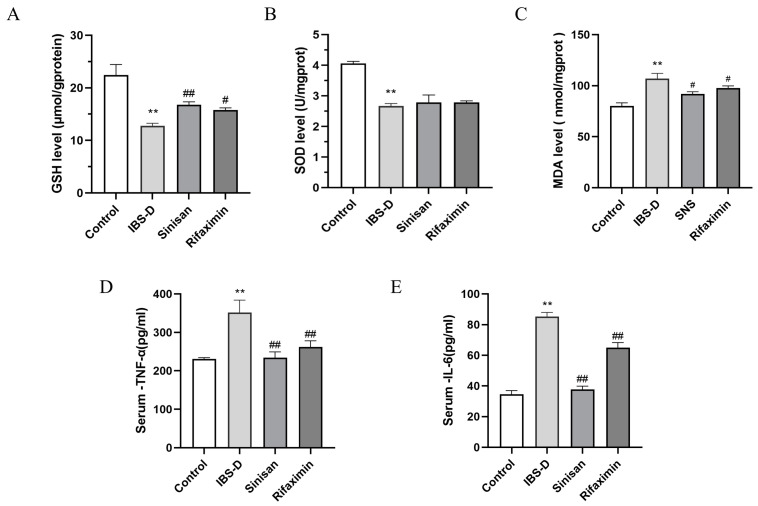
Effect of sinisan on serum levels of inflammatory factors and oxidative stress in colonic tissues of IBS-D mice. (**A**) The change in GSH concentration in all groups. (**B**) The change in SOD concentration in all groups. (**C**) The change in MDA concentration in all groups. Effect of sinisan on TNF-α (**D**) and IL-6 (**E**) levels in serum of IBS-D mice. (*n* = 3), ** *p* < 0.01 compared with the control group. # *p* < 0.05, ## *p* < 0.01 compared with the IBS-D group.

IBS-D is usually associated with abnormal changes in neurotransmitters, low-grade inflammation and oxidative stress levels. We examined some oxidative stress indexes. As shown in Figure 5A–C, the IBS-D group shows a significant lower level of antioxidative indexes Glutathione (GSH) and Super Oxide Dismutase (SOD) and a higher level of Malondialdehyde (MDA) content in colon tissues compared with the control group. Sinisan and rifaximin treatment can alleviate IBS-D induced-oxidative damage. In addition, sinisan and rifaximin reduced the elevation in Interleukin 6 (IL-6), and tumor necrosis factor-α (TNF-α) in serum. The levels of TNF-α and IL-6 are two important pro-inflammatory cytokines in the serum. Compared with the control group, the IBS-D group significantly increased the levels of TNF-α and IL-6 in serum. The application of sinisan and rifaximin can significantly lower the levels of TNF-α and IL-6 (Figure 5D,E). These results indicate that sinisan and rifaximin can effectively decrease serum inflammatory response and colonic oxidative damage.

### 2.5. Sinisan Ameliorates 5-HT and c-Fos in Brain Tissue of IBS-D Mice, Restores Up-Regulation of OCLN and ZO1 Expression in the Intestine of IBS-D Mice

**Figure 6:**
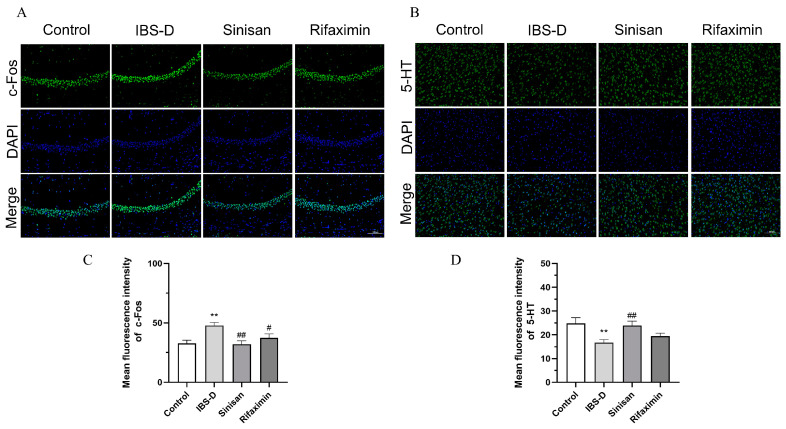
Immunofluorescence analysis of sinisan in hippocampal and prefrontal cortical tissues of IBS-D-induced depressive mice. (**A**) Representative images of c-Fos expression in the hippocampus (magnification 200×, scale bar = 100 μm). (**B**) Representative images of 5-HT expression in prefrontal cortex(magnification 200×, scale bar = 100 μm). (**C**) Mean fluorescence intensity of c-Fos. (**D**) Mean fluorescence intensity of 5-HT. (*n* = 3), ** *p* < 0.01 compared with the control group. # *p* < 0.05, ## *p* < 0.01 compared with the IBS-D group.

IBS-D is associated with c-Fos visceral hypersensitivity and 5-HT central emotional activity. We explored the effects of sinisan in IBS-D mice via the levels of c-Fos in the hippocampus and 5-HT in prefrontal cortical areas. As Figure 6A–D results show, IBS-D mice showed a significant elevation in c-Fos and decrease in 5-HT in fluorescence intensity compared with the control group. Sinisan treatment can significantly increase 5-HT levels and decrease c-Fos content, suggesting that sinisan is effective in regulating and improving IBS-D-induced depressive behaviors.

**Figure 7:**
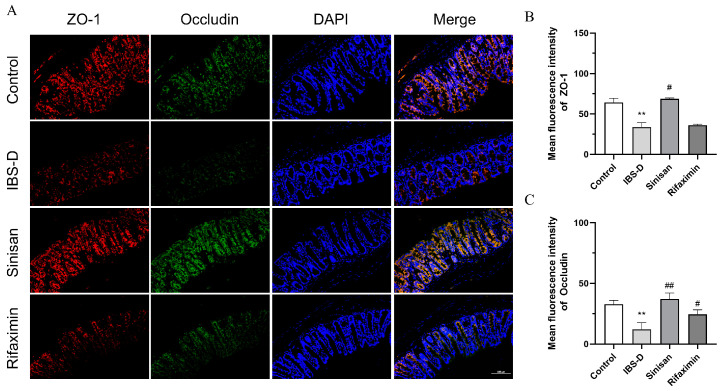
Sinisan increases expression of tight junction proteins in IBS-D mice. (**A**) Immunofluorescence micrograph (magnification 200×, scale bar = 100 μm) and its average absorbance value. (**B**) Mean fluorescence intensity of ZO-1. (**C**) Mean fluorescence intensity of occludin. (*n* = 3), ** *p* < 0.01 compared with the control group. # *p* < 0.05, ## *p* < 0.01 compared with the IBS-D group.

IBS-D is usually accompanied by increased intestinal mucosal permeability, and ZO-1 and occludin are key transmembrane proteins affecting the permeability of the tight junctions of colon tissues. We further validated the expression levels of tight junction proteins (TJs) in colonic tissues. As shown in Figure 7, the fluorescent expression of ZO-1 and occludin were significantly reduced in IBS-D colon tissues compared with the control group, suggesting that the intestinal mucosal barrier function was impaired in IBS-D mice. Fluorescence staining revealed that sinisan treatment increased the positive area of ZO-1 and occludin in the colonic tissues, suggesting that sinisan significantly enhanced the intestinal mucosal barrier function.

### 2.6. Sinisan Inhibits NF-κB Activation and Regulates Tlr4/Myd88/NF-κB Signaling Pathway in IBS-D Mice

Low-grade inflammation and immunological changes play important roles in the development of IBS-D. The NF-κB signaling pathway regulates innate and adaptive immune responses and anti-inflammatory cytokines. We examined the TLR4/Myd88/NF-κB pathway-related proteins and compared the mRNA expression levels of downstream key inflammatory cytokines such as TNF-α, IL-1β, and IL-6 by qRT-PCR.

**Figure 8:**
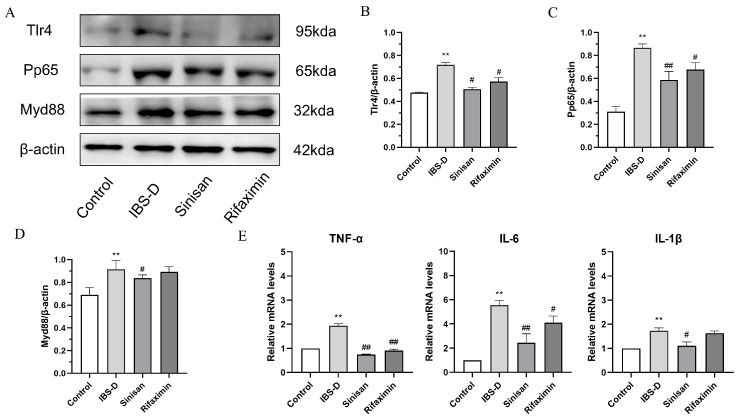
Sinisan regulates NF-κB expression in the colon of IBS-D mice. (**A**) Western blotting expression of Tlr4, pp65 and Myd88 expression in the colon of different groups of mice. (**B**) Tlr4/β-actin ratio. (**C**) Pp65/β-actin ratio. (**D**) Myd88/β-actin ratio. (**E**) mRNA expression levels of TNF- α, IL-6 and IL-1β in colon tissue were determined using RT-PCR. (*n* = 3), ** *p* < 0.01 compared with the control group. # *p* < 0.05, ## *p* < 0.01 compared with the IBS-D group.

To verify the function of sinisan on the TLR4/Myd88/NF-κB pathway, we detected the related proteins, including TLR4, MyD88, and pp65. As represented in Figure 8, compared with the control group, the levels of TLR4, MyD88, and pp65 were elevated in the IBS-D group mice. Sinisan treatment significantly suppressed these protein levels. Therefore, our study shows that sinisan can down-regulate the protein levels of the TLR4/Myd88/NF-κB pathway in vivo. Sinisan may indirectly reduce intestinal permeability by inhibiting the production of pro-inflammatory cytokines. qRT-PCR results showed that the IBS-D group had elevated higher levels of TNF-α, IL-1β, and IL-6 compared with the control group. Whereas, the expression of TNF-α, IL-1β, and IL-6 were significantly lower after sinisan treatment.

## 3. Discussion

IBS-D is a condition that disrupts the normal functioning of the intestinal tract, and interest in its research has grown in recent years. Nevertheless, the precise mechanisms behind IBS-D are still not fully understood. Several studies suggest that psychological factors and stress may play significant roles in the development of the disorder [20,21]. Current treatments include medications, dietary modifications, and psychotherapy, but most have side effects, making it especially important to find safer and more effective medications. Sinisan is a renowned Chinese herbal formula with a rich history of clinical application in TCM. Comprising *Bupleurum chinense DC*, *Paeonia lactiflora Pall*, *Citrus aurantium L.* and *Glycyrrhiza uralensis Fisch*, it is recognized for its effectiveness in smoothing the liver and regulating the spleen. This formula has been utilized in clinical settings for the treatment of gastrointestinal and emotional disorders for over 1800 years [20]. Clinically, sinisan is mostly used to treat depression and anxiety disorders that are mainly caused by liver depression [22]; it has also shown remarkable efficacy in the treatment of spleen and stomach disorders, effectively relieving abdominal pain and diarrhea and helping patients to restore the balance of the digestive tract [23]. According to Chinese medicine theory, IBS is mainly caused by liver depression and spleen deficiency. A variety of factors, such as emotional depression or prolonged stress, can lead to liver qi stagnation, which triggers liver depression, which in turn affects the normal functioning of the spleen, manifesting in gastrointestinal symptoms such as bloating and loose stools. This view is closely related to the modern medical theory of brain–gut interactions for IBS. However, studies on the pharmacological effects and mechanisms of sinisan in the treatment of IBS-D are still relatively limited. Therefore, this study aimed to investigate the therapeutic effect of sinisan on stress-induced IBS-D.

Currently, the main methods used to establish animal models of IBS-D include neonatal and maternal separation, restraint, water immersion restraint, chronic unpredictable mild stress, and stimulation with chemicals such as senna and acetic acid [7,24]. Acetic acid has long been regarded as an effective inducer for modeling recovery after experimental inflammation in order to study the pathophysiological mechanisms of IBS-D as well as the mechanism of action and efficacy of drugs. Therefore, the present study was conducted to establish an IBS-D mouse model by combining acetic acid enemas and restraint stress. Increased visceral sensitivity is regarded as an important clinical feature of IBS-D patients, and by using the AWR score, we found that the visceral pain threshold of IBS-D mice was significantly reduced, and sinisan was effective in increasing this threshold. In addition, IBS-D mice had significantly increased diarrhea and fecal water content, while both sinisan and rifaximin improved the diarrhea symptoms in the mice. Most IBS patients suffer from psychological disorders caused by stress, anxiety and depression. IBS-D mice treated with acetic acid enemas and restraint stress also exhibited anxiety- and depression-like behaviors. We analyzed the depression and anxiety-like behaviors using SPT, TST and OFT. The results showed that IBS-D mice had reduced sugar and water preference, decreased intermediate dwell time and distance traveled, prolonged immobility time, and exhibited significant depressive-like behaviors. HE staining showed that sinisan improved the neurological damage to the brain caused by IBS, as well as the low-grade inflammation observed in the intestines. Notably, sinisan was superior to rifaximin in improving depression-like behavior in the brain.

Intestinal tight junction proteins have an important role in IBS-D, and these include the transmembrane proteins (OCLN) and peripheral membrane proteins (ZO-1) [25]. Studies have shown that patients with clinical IBS-D have higher intestinal permeability than healthy individuals, and the increased permeability of the intestinal barrier makes it easier for inflammatory cells, bacteria, pathogens, and other antigens to invade, which triggers alterations in the intestinal ecosystem that pose a serious threat [26]. Tight junction proteins regulate the permeability of the intestinal barrier by tightly sealing the connections between cells, thus enhancing the function of the intestinal barrier [27], Histological assessment of inflammatory cells and changes in closure proteins and transmembrane proteins revealed that sinisan treatment was able to reduce inflammatory cells in colonic tissues while increasing the expression of closure proteins, thereby improving the barrier function of the colonic mucosa. In addition, sinisan significantly increased the brain levels of 5-HT neurotransmitters and C-fos proteins, which play important roles in gastrointestinal motility, visceral sensitivity, and stress and mood regulation [28,29,30].

Inflammatory factors and oxidative stress status are important indicators for the diagnosis of IBS-D [31,32]. In our model, it was observed that sinisan treatment was able to reduce serum levels of IL-6 and TNF-α, as well as levels of SOD, GSH, and MDA in colonic tissues, thus reversing oxidative stress. This result is consistent with the alleviation of systemic inflammatory response in IBS-D mice.

Low-grade inflammation is considered a key factor in IBS [33,34]. A growing body of research suggests that some patients with IBS have low-grade inflammation in the colon, and that suppressing and reducing intestinal inflammation can help with disease recovery [31,34,35]. Some of the major components in sinisan have demonstrated significant therapeutic effects in animal models of IBS by modulating inflammation-related pathways. For example, chaihu saponins were able to modulate inflammation, oxidative stress, and the brain–gut axis [36], Hesperidin, on the other hand, increases antioxidant expression and inhibits NF-κB activation and inflammatory cytokine secretion by activating the PI3K/AKT-Nrf2 pathway in the liver [37]. Paeoniflorin has a wide range of biological activities and can exert anti-inflammatory, antioxidant and immunomodulatory effects through the TLR4-NF-κB signaling pathway [38], and it is also used in depression and neurological disorders [39]. Glycyrrhizin, on the other hand, attenuates the inflammatory response by inhibiting NF-kB, p38/ERK pathway and cellular pyroptosis [40], These findings suggest that sinisan may alleviate IBS-D through related inflammatory pathways. As a pattern recognition receptor, TLR4 plays a key role in mucosal immune responses, and its activation may trigger excessive inflammatory responses, impair intestinal barrier function, and promote the development and progression of related diseases. Studies have shown that the activation of TLR4 is strongly associated with IBS [41,42]. TLR4 activates the NF-κB signaling pathway through the MyD88 pathway and promotes the production of inflammatory factors TNF-α and IL-6 [43,44]. NF-κB, as an important intracellular transcription factor, is involved in the regulation of inflammation and immune responses [45]. TLR4 activates the NF-κB pathway through MyD88, leading to increased expression of pro-inflammatory factors [46], and NF-κB activation may play an important role in stress, intestinal barrier function, and microbiota interactions, thereby increasing the number of immune cells and chemokines, leading to increased intestinal permeability. qRT-PCR results showed that the levels of pro-inflammatory cytokines were significantly elevated in the IBS-D mouse model but significantly decreased after sinisan treatment, suggesting that sinisan was able to inhibit NF-κB signaling and thus regulate intestinal permeability. Our study demonstrated that sinisan significantly alleviated the symptoms of IBS-D and ameliorated intestinal dysfunction and depression-like behavior in mice, which may be achieved by inhibiting the TLR4/MyD88/NF-κB pathway.

There are some limitations in this study. First, there is a lack of validation of in vitro experiments and in-depth exploration of the pathways related to sinisan’s action in the brain. In addition, the specific role of sinisan in brain–gut inter-regulation is unclear. Although several studies have emphasized the importance of sinisan’s main components (e.g., hesperidin, isoglycyrrhizin, glycyrrhizin, paeoniflorin, and shibaosaponin A) in antidepressant and restoration of colonic barrier functions, the present study did not analyze the therapeutic efficacy of these specific components in depth in the context of acetic acid-bound stress-induced IBS-D. Although an association was observed between sinisan treatment of IBS-D and depressive-like behaviors and colonic damage, the broader effects of sinisan and its relationship to other variables require further study. Future studies will focus on the mechanism of action of sinisan and its different active ingredients in IBS-D brain–gut interactions and validate the interactions between sinisan and IBS-D through the use of agonists or antagonists to reveal the modulation of the relevant signaling pathways and to identify the potential active ingredients of sinisan. These studies will provide a more solid theoretical foundation for the clinical application of sinisan in the treatment of IBS-D and open up new directions for the study of brain–gut axis-based IBS therapy.

## 4. Materials and Methods

### 4.1. Chemicals and Reagents

Rifaximin was purchased from Sichuan Baili Pharmaceutical Co., Ltd. (Chengdu, China) and dissolved in saline for intragastric administration. Acetic acid was obtained from Phygene (Fuzhou, China). Isoflurane was purchased from Hebei Jindafu Pharmaceutical Co. (Shijiazhuang, China) Oxidative stress kit was purchased from Nanjing Jiancheng (Nanjing, China). Propylene glycol (MDA) assay kit (TBA method), No. A003-1-2; SOD kit (WST-1 method), No. A001-3-2; reduced glutathione (GSH) assay kit (microplate method), No. A006-2-1. Il-6 Eisa kit was purchased from Quanzhou Ruixin Biotechnology Co., Ltd. (Quanzhou, China), Item No. 2024051577701, and TNF-α Elisa kit was purchased from Wuhan Fiyue Bio-technology Co., Ltd. (Wuhan, China), Item No. E24OKY947.

### 4.2. Phytomedicine and Preparation of Sinisan

Sinisan consists of four raw herbs: *Bupleurum chinense DC* (Apiaceae), *Paeonia lactiflora Pall* (Paeoniaceae), *Citrus aurantium L.* (Aurantioideae) and *Glycyrrhiza uralensis Fisch* (Fabaceae), Chinese medicinal herbs were purchased from Beijing Tongrentang Guangzhou Pharmaceutical Chain Co., Ltd. (Guangzhou, China), as shown in Table 1 and the Sinisan was prepared in accordance with the method described in the Chinese Pharmacopoeia in 2020, according to the previous preparation method [19]. After adding 12 times the amount of water to infuse for 1 h, sinisan was extracted twice by water decoction, filtered and the two filtrates combined, concentrated to the equivalent of the original liquid 1.0 g/mL, diluted to 0.624 g/mL, and used for intragastric administration.

### 4.3. HPLC-MS Analysis of Sinisan

A measure of 0.1 g of sinisan extract was weighed, 1 mL of 80% methanol was added and ground using grinding beads for 5 min, then vortexed and mixed for 10 min, followed by centrifugation at 13,000 rpm for 10 min, and finally the supernatant was taken for analysis. The chromatographic column was an AQ-C18, 150 mm × 2.1 mm, with a particle size of 1.8 µm, and the flow rate was set at 0.30 mL/min. The aqueous phase was 0.1% formic acid aqueous solution, and the organic phase was methanol. The column temperature was maintained at 35 °C, the autosampler temperature was set at 10.0 °C, and the injection volume was 5.00 µL.

### 4.4. Animals and Treatments

In this study, we used 6- to 8-week-old adult female C57BL/6 mice. Mice were housed in standard mouse cages with the ambient temperature controlled at 24 ± 2 °C and humidity maintained at 50%–60%. Mice had access to water and food at all times, and the light and dark cycles alternated for 12 h.

To construct the IBS-D model in mice, we first lightly anesthetized the mice with isoflurane. After successful anesthesia, 0.15 mL of 4% acetic acid was instilled into the colon at 2 cm proximal to the anus for 30 s. The mice were then turned upside down for 10 s to prevent leakage of the solution, followed by an additional instillation of 0.15 mL of phosphate-buffered saline (PBS) to dilute the acetic acid and to flush the colon. Mice in the blank group were treated in the same way as the other groups, but perfused with 0.15 mL of PBS instead of 4% acetic acid. Next, we wrapped the upper forelimbs and thoracic trunks of the mice with medical tape to apply restraint pressure, and the restraint was applied at a level that ensured that it did not impede the mice’s locomotion. Mice received 2 h of restraint pressure per day for one week. After confirming the successful establishment of the mouse IBS model, no further modeling treatments were performed, allowing the mice to have a recovery period prior to administration of the treatment. Mice in the control group were instead perfused with 0.1 mL of saline and were not subjected to restraint pressure. Mice were randomly divided into groups (n = 6) based on body weight: control group, IBS-D group, rifaximin group (10 mg/kg/day), and sinisan group (6.24 g/kg/day). The mice in the sinisan and rifaximin groups received daily gavage treatment with the corresponding drugs and doses, whereas the mice in the control and IBS-D group were treated by gavage using an equal volume of saline, administered twice daily for 5 consecutive days.

### 4.5. Behavioral Tests

#### 4.5.1. Body Weight and Fecal Water Content

During the experiment, we monitored the body weight of the mice and determined the moisture content by collecting and weighing the feces of each mouse. Samples were dried in an oven at 60 °C for 24 h and their weights were recorded. The moisture content of the feces was calculated indirectly through the loss of fecal weight using the formula [(initial weight − weight after drying)/initial weight] × 100%.

#### 4.5.2. Visceral Hypersensitivity Assessment

After model establishment and completion of drug administration, the colorectal distension test was used to assess the visceral sensitivity of mice in each group. The mice were first lightly anesthetized and then lubricated with paraffin oil, followed by gentle insertion into the anus of the mice using a 6FR pediatric Foley catheter (Yixin Medical Devices Co., Ltd., Shanghai, China) with a balloon. After the mice were awake and acclimatized, balloon dilatation was performed by injecting different volumes of air into the balloon and held for 20 s. Dilatation was performed at 4 min intervals and repeated a total of three times to ensure the accuracy of the results. Behavioral tests were performed under blinded conditions [47].

#### 4.5.3. Sucrose Preference Test

The SPT is often used as an indicator to assess pleasure deficit, which is a major feature of depression. On the first day, two bottles of 100 mL each of 2% sucrose water were placed in the mouse cages. On the second day, one bottle of 2% sucrose water was kept unchanged and the other bottle was replaced with the same volume of pure water. By the third day, the mice were exposed to both a 100 mL bottle of 2% sucrose solution and a bottle of the same volume of purified water, and the position of the water bottles was changed after 2 h in the experiment in order to avoid developing a positional preference. Sucrose preference was assessed by calculating the percentage of total sucrose solution consumed.

#### 4.5.4. Open Field Test

The OFT is capable of quantitatively assessing the animals’ spontaneous activity, exploratory behavior, and anxiety and depressive states, and is an important indicator for assessing depression. The experiment was conducted in a field measuring 30 × 30 × 30 cm^3^ with color-coded mouse dividers and placed in a dimly lit isolation room. For behavioral testing, each mouse was placed in the center of the field and allowed to move freely. Subsequently, the autonomous exploratory behavior of the mice was recorded by a camera fixed above the venue and connected to computer software, and the distance they moved and the time they stayed in the central area were analyzed. Each mouse was wiped down with 75% alcohol before testing to wipe down the experimental box and feces to avoid any effect on the next mouse.

#### 4.5.5. Tail Suspension Test

The TST is a classic and valid depression assessment tool. In the test, a mouse’s tail is secured with tape so that its head is pointed down and at a distance from the ground. During the test, the suspended mice first struggle or exhibit escape behavior a few times, and then remain temporarily still. We quantified the duration of immobility of the mice during the first few minutes [48].

### 4.6. Measurement of Oxidative Stress Indicators

Oxidative stress is a state of imbalance between oxidative and antioxidant mechanisms in the body and is closely associated with the development of IBS-D. At the end of animal sampling, the frozen colon tissue was removed from −80 °C refrigerator and cut into 1 cm lengths, and processed accordingly according to the kit instructions in order to detect the content of oxidative index MDA as well as antioxidative indexes SOD and GSH in the colon.

### 4.7. Enzyme-Linked Immunosorbent Assay (ELISA)

After the mice were anesthetized with isoflurane, the eyes were removed for blood collection. The blood samples were allowed to stand at room temperature for 30 min, and then centrifuged at 3000 rpm for 15 min to separate the serum. Next, TNF-α and IL-6 levels in each group of sera were detected using an ELISA kit according to the manufacturer’s instructions.

### 4.8. Histologic Evaluation of Hematoxylin-Eosin Staining and Immunofluorescence Correlations

After the mice were executed, cardiac perfusion was first performed to extract brain tissue from the mice, and then approximately 1 cm segments of descending colon tissue were cut. Next, the brain and colon tissues were fixed in paraformaldehyde solution, followed by dehydration and embedding in paraffin, and cut into 4 μm slices. After that, the slides were dried, deparaffinized and rehydrated, and stained with hematoxylin-eosin (HE) solution. In immunofluorescence (IF) experiments, paraffin sections of brain and colon tissues were incubated with primary antibodies (occludin and ZO-1, 1:100) overnight at 4 °C, followed by incubation with fluorescent secondary antibodies for 1 h in the dark. Sections were restained with DAPI and visualized using a high-performance fluorescence microscope (BX51-32FL, Olympus, Hachioji-shi, Japan). The acquired images were merged and processed by ImageJ software 1.54k.

### 4.9. Western Blot Analysis

Colon tissue from each group was removed from the refrigerator at −80 °C, separated using scissors and weighed (approximately 30 mg). Subsequently, 200 μL of RIPA buffer containing phosphatase and protease inhibitors was added to each sample, and the total proteins of the corresponding colon tissues were extracted by homogenization, sonication, and centrifugation. The collected samples were quantitatively analyzed for protein concentration using the BCA protein assay kit. The samples were loaded onto an SDS-PAGE gel for electrophoresis, and then the proteins were transferred to a 0.45 μm PVDF membrane. The membranes were closed with rapid closure solution for 20 min at room temperature and incubated with primary antibody at 4 °C overnight. The antibodies used were diluted in the following ratios: anti-Tlr4 (1:1000), anti-Myd88 (1:1000), anti-NF-κB (1:1000) and anti-β-actin (1:10,000). After rewarming the next day, the membranes were washed and incubated with the appropriate secondary antibodies. After being configured using an ultrasensitive luminescent solution at a 1:1 ratio, the strips were immersed and the corresponding protein expression was photographed using an ImageQuant LAS4000 luminescent biomolecular imager (Shanghai, China). Finally, the grayscale values of the strips were analyzed using ImageJ software and compared with the grayscale values of the internal reference β-actin strips.

### 4.10. Quantitative Reverse Transcription Polymerase Chain Reaction (Q-RTPCR)

RNA was extracted from colon tissues using TRIzol reagent and cDNA was synthesized according to the manufacturer’s instructions. mRNA levels were subsequently determined using SYBR Green Mix by Roche’s LightCycler 96 (Shanghai, China) real-time fluorescent quantitative PCR system. The 2^−ΔΔCt^ method was used to calculate the relative mRNA expression levels of each sample tissue, and the relevant primer information is shown in Table 2.

### 4.11. Statistical Analysis

Statistical analyses were expressed as mean ± standard error of mean (SEM), and one-way analysis of variance (ANOVA) was performed to statistically test multiple sets of data using Microsoft Excel 2021 and GraphPad Prism 9.0 software. In all cases, differences were considered statistically significant when the *p*-value was <0.05.

## 5. Conclusions

Taken together, our findings suggest that sinisan ameliorates mucosal inflammatory responses during IBS-D, upregulates the expression of OCLN and ZO-1 in colonic tissues, reverses the decrease in 5-HT and the elevation in c-Fos to enhance intestinal barrier function and ameliorates depressive-like behavior in mice. These findings confirm that sinisan is an effective therapeutic strategy for the treatment of IBS-D to improve intestinal permeability and restore intestinal mucosal barrier function by inhibiting the NF-κB signaling pathway.

## Figures and Tables

**Table 1:** Herbal components of sinisan.

Plant Name	Chinese Name	Place of Origin	Voucher Number
*Bupleurum chinense DC*	Chaihu	Hebei, China	22120101
*Paeonia lactiflora Pall*	Baishao	Anhui, China	22060801
*Citrus aurantium L.*	Zhishi	Jiangxi, China	22030601
*Glycyrrhiza uralensis Fisch*	Gancao	Inner Mongolia, China	22030502

**Table 2:** The q-PCR primer sequences used in this study.

Name	Forward	Reverse
***TNF-α***	CGGGCAGGTCTACTTTGGAG	ACCCTGAGCCATAATCCCCI
***IL-6***	CTGCAAGAGACTTCCATCCAG	AGTGGTATAGACAGGTCTGTTGG
***IL-1β***	AATCTCGCAGCAGCACATCA	GGAAGGTCCACGGGAAAGAC
***β-actin***	GCTTCTTTGCAGCTCCTTCG	ACCCATTCCCACCATCACAC

## Data Availability

Data will be made available on request.

## Supplementary Materials

The supporting information can be downloaded https://www.mdpi.com/article/10.3390/ijms251910262/s1.
